# Estimating plant abundance using inflated beta distributions: Applied learnings from a lichen–caribou ecosystem

**DOI:** 10.1002/ece3.2625

**Published:** 2016-12-20

**Authors:** Jonah L. Keim, Philip D. DeWitt, J. Jeremy Fitzpatrick, Noemie S. Jenni

**Affiliations:** ^1^ Matrix Solutions Inc.EdmontonABCanada; ^2^ Ontario Ministry of Natural Resources & ForestryScience and Research BranchPeterboroughONCanada

**Keywords:** beta regression, boreal forest, fruticose lichens, LiDAR, proportional data, satellite imagery, woodland caribou, zero‐one‐inflated distributions

## Abstract

Quantifying abundance and distribution of plant species can be difficult because data are often inflated with zero values due to rarity or absence from many ecosystems. Terrestrial fruticose lichens (*Cladonia and Cetraria* spp.) occupy a narrow ecological niche and have been linked to the diets of declining caribou and reindeer populations (*Rangifer tarandus*) across their global distribution, and conditions related to their abundance and distribution are not well understood. We attempted to measure effects related to the occupancy and abundance of terrestrial fruticose lichens by sampling and simultaneously modeling two discrete conditions: absence and abundance. We sampled the proportion cover of terrestrial lichens at 438 vegetation plots, including 98 plots having zero lichens. A zero‐inflated beta regression model was employed to simultaneously estimate both the absence and the proportion cover of terrestrial fruticose lichens using fine resolution satellite imagery and light detection and ranging (LiDAR) derived covariates. The probability of lichen absence significantly increased with shallower groundwater, taller vegetation, and increased *Sphagnum* moss cover. Vegetation productivity, *Sphagnum* moss cover, and seasonal changes in photosynthetic capacity were negatively related to the abundances of terrestrial lichens. Inflated beta regression reliably estimated the abundance of terrestrial lichens (*R* ^2^ = .74) which was interpolated on a map at fine resolution across a caribou range to support ecological conservation and reclamation. Results demonstrate that sampling for and simultaneously estimating both occupancy and abundance offer a powerful approach to improve statistical estimation and expand ecological inference in an applied setting. Learnings are broadly applicable to studying species that are rare, occupy narrow niches, or where the response variable is a proportion value containing zero or one, which is typical of vegetation cover data.

## 1 Introduction

Organisms that are rare or occupy narrow ecological niches are infrequently observed in nature. As a result, randomly or systematically collected data on the habitat use, abundance and distribution of such species are commonly overdispersed with zero values (Welsh, Cunningham, Donnelly, & Lindenmayer, 1996). Guiding sampling efforts to solely obtain occurrence data or failing to account for zero‐inflation can lead to incorrect ecological inferences (Martin et al., 2005). Several statistical methods accommodate zero‐inflated data, including the zero‐inflated Poisson (Lambert, 1992), zero‐inflated negative binomial (Hall, 2000), and hurdle (Mullahy, 1986) models for count data, and the zero‐ and one‐inflated beta distribution model (Ospina & Ferrari, 2010) for proportional data. These models enable the simultaneous estimation of multiple components in a statistical distribution by accommodating zero‐inflation (e.g., zero‐inflated Poisson, zero‐inflated negative binomial) or a condition of absence (e.g., hurdle model, zero‐inflated beta). Sampling for absence and abundance and employing models that accommodate zero‐inflation enable scientists to infer effects on both the absence and abundance of a species. We demonstrate the value of this approach to support the conservation and reclamation of terrestrial fruticose lichens and woodland caribou habitat in the boreal forest.

**Figure 1:**
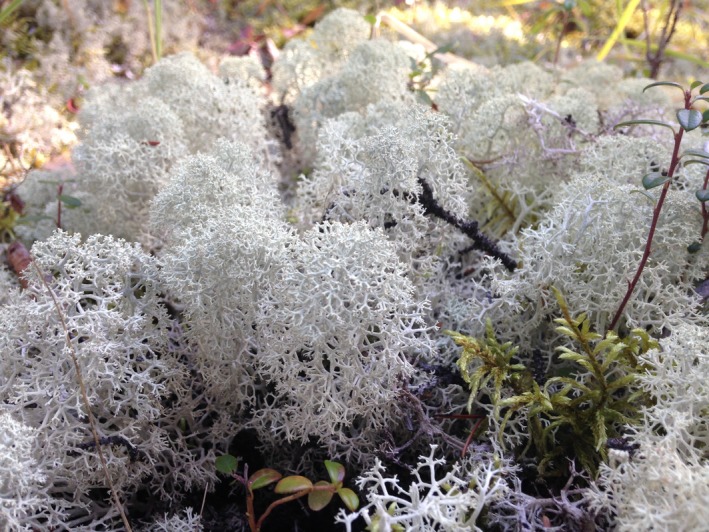
Photograph of *Caldonia stellaris* taken in the boreal forest of Alberta, Canada, during the vegetation survey conducted for this study. Photograph credit: J. Jeremy Fitzpatrick

Terrestrial fruticose lichens from the genera *Cladonia and Cetraria* (Figure 1) occupy a narrow ecological niche in boreal ecosystems and are primary forages for caribou and reindeer (*Rangifer tarandus*) across their global distribution (Baskin, 1986; Kojola, Helle, Niskanen, & Aikio, 1995; Schaefer & Pruitt, 1991; Thompson et al., 2015). They generally occur in mature forests with nitrogen‐poor substrates and elevated acidity, such as sand or dry peat soils (Kershaw, 1985; Morneau & Payette, 1989; Appendix S1). Lichens must be wetted to photosynthesize (Kershaw, 1972; Lechowicz & Adams, 1974) but do not depend on soils for moisture because they can absorb moisture from the air (Kershaw, 1985). Thus, at local scales, terrestrial forage lichens tend to occur where soils are too shallow or sterile to support vascular plants, and microclimate conditions are cool and humid enough to facilitate growth (Kershaw, 1985; Nash, 1996). However, the combination of conditions that promote lichen establishment and abundance is not completely understood, in part, because few studies have evaluated the relationship between lichen absence, abundance, and the numerous conditions that occur in nature. The fact that lichen growth rates are sensitive to substrate characteristics and microsite humidity suggests that fine‐scale data may be needed to accurately estimate multivariate relationships with lichen abundance.

Ecosystems that contain terrestrial and arboreal *Bryoria, Alectoria,* and *Usnea* forage lichens have been linked to the distribution, diet, and health of caribou which are declining throughout much of their global distribution (Vors & Boyce, 2009). In recent years, there has been increased focus on woodland caribou conservation in Canada where caribou populations appear to be declining due to the combination of human disturbance, low reproduction, predation, and habitat loss (Environment Canada 2012; Sorensen et al., 2008). Observational data reveal that woodland caribou select forest stands with increased abundances of lichens when foraging (Appendix S2; Johnson, Parker, & Heard, 2000) and that lichens comprise over 65% of their winter diet (Thompson et al., 2015). Recent research has linked fecal progesterone to the abundance of lichens in caribou ranges and the diet of female caribou during gestation (Keim, Wasser, Lele, DeWitt, & Taper, 2012), suggesting that caribou demography is related to lichen intake.

Despite the linkages between forage lichens and woodland caribou, there remain knowledge gaps regarding the ecological conditions that support lichens, the abundance and distribution of lichens, and methods to recover lichens following human disturbances. There are few examples where the spatial distribution of terrestrial lichen abundance has been estimated under forested conditions; however, their distribution has been estimated at broad spatial scales using multispectral reflectance data from Landsat and SPOT satellites in tundra and alpine ecosystems (Käyhkö & Pellika, 1994; Nordberg & Allard, 2002; Thompson, Klassen, & Cihlar, 1980).

We conducted a study to delineate terrestrial forage lichens and identify ecological conditions that support their abundance in the boreal forest. Our study had two objectives. First, develop a sampling design and analysis approach to delineate the geographic distribution and abundance of terrestrial forage lichens. Second, measure and better understand the ecological conditions that support terrestrial forage lichens to improve their reclamation. We had previously sampled a subset of lichen‐abundant boreal ecosystems to better understand their ecological associations. We found that terrestrial forage lichens occur within specific ecological conditions defined by poor soil fertility (pH of 3.5–5; carbon–nitrogen between 30:1 and 80:1) and mature conifer forests (>50 years; Appendix S1). We inferred that studying conditions related to both the abundance and absence of terrestrial forage lichens could provide additional insights on their distribution, abundance, and reclamation. Recent statistical developments allow the estimation of inflated distributions, such as the beta distribution which is otherwise constrained between zero and one (Ospina & Ferrari, 2010). We therefore designed our study to sample a wide range of ecosystems found in the boreal forest, including those with zero lichen cover. Our research advances the ecological understanding of terrestrial forage lichens and the management of woodland caribou habitat and demonstrates how sampling for and estimating zero‐inflated distributions can improve statistical estimation and expand inference in ecological studies.

## 2 Materials and Methods

Our study was conducted within the Athabasca oil sands in Alberta, Canada. It is located within an area managed for both timber and oil extraction in the West Side Athabasca River (WSAR) caribou herd range. Active oil exploration and development using seismic and drilling techniques, steam‐assisted gravity technologies, and surface mining are present in and surrounding our study area. Industrial activities have grown rapidly in the past decade and, as a result, there is a need for cost‐effective and timely management tools that can be used to maintain and recover woodland caribou habitat (Environment Canada 2012). Complimentary studies on woodland caribou habitat use and habitat restoration are underway in our study area.

The study area is dominated by boreal wetlands defined by organic peat soils (i.e., peatlands) and flat topographic conditions. Vegetation composition is influenced by soil moisture. Drier peat soils are characterized by black spruce (*Picea mariana*) trees with low vascular plant abundance while wetter soil ecosystems transition toward tamarack (*Larix laricina*) trees or shrub fens with increased vascular plant abundance. Wildfire is a dominant ecological process in this ecosystem and influences the abundance and distribution of terrestrial forage lichens (Dunford, McLoughlin, Dalerum, & Boutin, 2006). A portion of our study area burned in 1982; the remainder has not experienced a wildfire since before 1930. The area is thus largely dominated by mature forest ecosystems wherein terrestrial forage lichens may occur.

### 2.1 Vegetation sampling

Our study focused on two ecological components in the boreal forest: peatland ecosystems and terrestrial forage lichens. Peatland ecosystems that are predominantly sheltered by black spruce have been tied to woodland caribou ranges and habitat use patterns (Rettie & Messier, 2000; Schaefer & Pruitt, 1991). Although common to caribou ranges in the boreal forest, not all black spruce peatlands contain lichens, the reasons for their presence and absence are often indistinct (Appendices S1 and S2), and there is considerable uncertainty in the ability to recover these ecosystems (Alberta Environment and Parks 2015). Second, the sampling design focused on terrestrial forage lichens from the genera *Cladonia* and *Cetraria*. We focused on terrestrial forage lichens because they comprise a larger portion of the woodland caribou diet than arboreal forage lichens (Schaefer & Pruitt, 1991; Thompson et al., 2015), the presence of terrestrial and arboreal forage lichens is often correlated (Appendix S1), and terrestrial forage lichens are coupled to the forest floor, rather than trees, making them more sensitive to remote‐sensing and reclamation applications than arboreal forage lichens.

In September 2014, we collected vegetation plot data along transects located within four geographic strata designed to balance sampling intensity across the study area. Sampling transects were typically box‐shaped to converge the sampling start and end points in each day of sampling. Transects were initiated from a random starting location and in a randomly selected cardinal direction. Transects followed an irregular box‐shape if environmental obstacles were encountered (e.g., rivers, beaver impoundments) or to accommodate safe egress options. Vegetation data were collected at two plot types: fixed and opportunistic plots. Fixed plots were sampled every 100 m along a systematic transect grid created a priori to ensure that the data were collected across a range of environmental conditions regardless of lichen presence. Opportunistic plots were sampled between fixed plots if crews encountered a lichen patch with >10% lichen cover and the lichen patch was at least 25 m from the previous plot. Collecting data at opportunistic plots allowed us to efficiently increase the sample size of lichen‐abundant plots.

Vegetation plots were defined by a 2 × 2 m quadrat. This size was chosen based on a previous inventory (Appendix S1) and because substrate and microsite conditions important to lichen growth rates (Kershaw, 1985) can vary at fine spatial scales. At each plot location, photographs were recorded along with data on the proportion cover of terrestrial forage lichens, graminoids, mosses, *Sphagnum* mosses, herbs/forbs, shrubs, water, and bare earth/litter. In addition, shrub height (m), and tree height (m) and species composition data were collected at each plot. Terrestrial forage lichens were not identified to species in the field. Based on detailed vegetation plots sampled in a neighboring location, the dominant species within the region was *Cladonia mitis*. Other terrestrial forage lichen species included *Cladona stygia*,* Cladonia stellaris*,* Cetraria nivalis*, and *Cladonia rangerifera* (Appendix S1).

### 2.2 Statistical analyses

Our response variable was the proportion of terrestrial forage lichens covering each vegetation plot (range: 0–0.85). We used proportion cover recognizing that it could be rapidly sampled in a field setting and that others have correlated lichen cover with lichen biomass (Dunford et al., 2006, *R* ^2^ = .95). Beta regression is often used to estimate proportional data constrained between zero and one (Ferrari & Cribari‐Neto, 2004) and has shown to provide unbiased parameter estimates for vegetation cover data (Eskelson et al. 2011). Recognizing that our response variable included zero values, we used a zero‐inflated beta regression model (Ospina & Ferrari, 2010). The zero‐inflated model estimates the beta distribution for values constrained between zero and one (Ferrari & Cribari‐Neto, 2004) and the probability of a zero measure (Ospina & Ferrari, 2010). Before and during model selection procedures, we explored the data and parameters to aid interpretation and identify patterns and anomalies in the data (e.g., correlations, skewed distributions). Statistical models were estimated using maximum‐likelihood methods in the GAMLSS‐package in R (Stasinopoulos & Rigby, 2007). Candidate models and covariates were selected using Akaike information criterion (Akaike, 1973).

We considered a combination of covariates derived from satellite imagery and light detection and ranging (LiDAR) spatial data sources. Three satellite imagery products available to map vegetation attributes at different spatial scales were considered as follows: Landsat 8 imagery provides information on deep blue, blue, green, red, near‐infrared, and short‐wave infrared reflectance at 30‐m spatial resolution; SPOT 6 imagery provides information on blue, green, red, and near‐infrared reflectance at 6‐m spatial resolution; and QuickBird imagery provides information on blue, green, red, and near‐infrared reflectance at 2.5‐m spatial resolution. We considered the raw reflectance bands and normalized difference vegetation index (NDVI) as a composite covariate. NDVI was calculated as (NIR − R)/(NIR + R) using the near‐infrared (NIR) and red (R) bands from each satellite imagery product. We also obtained Landsat 8 data from both mid‐summer and late‐fall and calculated the seasonal change in NDVI to differentiate locations whose photosynthetic capacity varies by season, such as areas of deciduous vegetation (high seasonal variation) and coniferous vegetation (low seasonal variation). LiDAR‐derived bare earth and a full feature digital elevation model were obtained from airborne imaging and available to our study at a spatial resolution (pixel size) of 1 m. The bare earth (surface) values were subtracted from the full feature digital elevation model to calculate a vegetation height covariate across our study area (Magnussen & Boudewyn, 1998). A LiDAR‐derived covariate for groundwater depth (minimum depth where spaces in the soil become saturated with water) was also calculated from bare earth and full feature LiDAR following methods in Murphy et al. (2011). We postulated that LiDAR‐derived covariates may help us differentiate terrestrial forage lichens from other vegetation should spectral confusion occur. As a final consideration, we used our data to develop a spatial covariate for *Sphagnum* moss cover (Supporting Information 3) because exploratory analyses of our field data revealed that terrestrial lichen cover was negatively correlated with the abundance of *Sphagnum* mosses (*R* = .51).

We plotted the residuals from the final model to assess whether errors in the model were correlated with spatial covariates for latitude, longitude, or sampling transect. We then evaluated the fit of the estimated model to ascertain its predictive ability by estimating the linear relationship between field measurements and model predictions. To illustrate the utility of predicting lichen cover, we spatially interpolated the estimated lichen model in a geographic information system and contrasted the spatial arrangement of lichen cover (estimated by the model) to areas of human disturbance that require future reclamation.

## 3 Results

Field crews sampled 438 vegetation plots (251 fixed and 187 opportunistic plots) across 23.9 km of survey transect. The vegetation plots were distributed across organic peat (*n* = 394) and mineral (*n* = 44) soil ecosystems in similar proportions to what was available across the study area. A large number of survey plots contained zero lichen cover (*n* = 98).

The final regression model estimating the proportion cover of terrestrial forage lichens (Table 1) indicates that lichen cover is related to a combination of ecological and remotely sensed variables. The final model includes covariates for blue reflectance, near‐infrared reflectance, seasonal change in NDVI, vegetation height, *Sphagnum* moss cover, depth to groundwater, and latitude–longitude. QuickBird imagery was better at predicting terrestrial forage lichen cover than Landsat 8 (AIC difference = −93.54) and SPOT 6 (AIC difference = −32.06) imagery products. This result suggests that finer resolution imagery (2.5 m) may help differentiate small patches of lichen whose reflectance values are averaged with other vegetation in coarser scale imagery such as SPOT 6 and Landsat 8.

**Table 1:** Parameter estimates in the zero‐inflated beta regression model for terrestrial forage lichen cover

Parameter	Estimate	*SE*	*t* Value	Pr (>|*t*|)
Beta Model (Proportion model)				
Intercept	−7.922	3.177	−2.494	.013
Seasonal change in NDVI	−20.394	5.124	−3.980	<.001
Blue^a^	0.074	0.027	2.714	<.001
Near‐infrared^a^	−4.438×10^−3^	2.063×10^−3^	2.123	.003
Vegetation height	−0.140	0.029	−4.780	<.001
*Sphagnum*	−2.306	0.598	−3.857	<.001
Northing	−0.490	0.064	−7.654	<.001
Easting	−0.209	0.059	−3.536	<.001
Sigma link function				
Intercept	12.438	2.373	5.241	<.001
Blue^a^	−0.093	0.021	−4.492	<.001
Near‐infrared^a^	5.212×10^−3^	1.469×10^−3^	3.549	<.001
Northing	0.441	0.084	5.259	<.001
Logit Model (zero‐inflation model)				
Intercept	−6.823	1.21	−5.629	<.001
Vegetation height	0.437	0.074	5.897	<.001
Seasonal change in NDVI	48.032	9.005	5.334	<.001
Easting	0.656	0.187	3.506	<.001
Depth to groundwater	−1.851	0.850	−2.177	.030
*Sphagnum*	7.227	1.494	4.838	<.001

a Reflectance values taken from QuickBird imagery.

Overall, the proportion of terrestrial forage lichen cover increased with blue reflectance and decreased with near‐infrared reflectance. Reflectance values reflect the combined vegetation characteristics within and surrounding the vegetation plot, suggesting that lichens are primarily found in vegetated areas (i.e., higher blue values) with low plant productivity (i.e., lower near‐infrared values). In combination, the seasonal change in NDVI, vegetation height, and *Sphagnum* moss cover influenced terrestrial lichen cover in two ways: (1) The proportion of terrestrial lichen decreases as the seasonal change in NDVI, vegetation height, and *Sphagnum* moss cover increased; and (2) the probability of observing zero terrestrial lichens increases as the seasonal change in NDVI, vegetation height, and *Sphagnum* moss cover increased. These results are consistent with the understanding that boreal ecosystems with evergreen vegetation contain more terrestrial lichen cover than locations with increased deciduous and shrub vegetation (e.g., *Betula*,* Salix* and grasses); that terrestrial lichen cover is negatively correlated with *Sphagnum* mosses; and that terrestrial lichen cover is more abundant in areas with shorter vegetation. Results show that areas with >20% terrestrial lichen cover occur in areas with vegetation heights that are shorter than 7 m.

**Figure 2:**
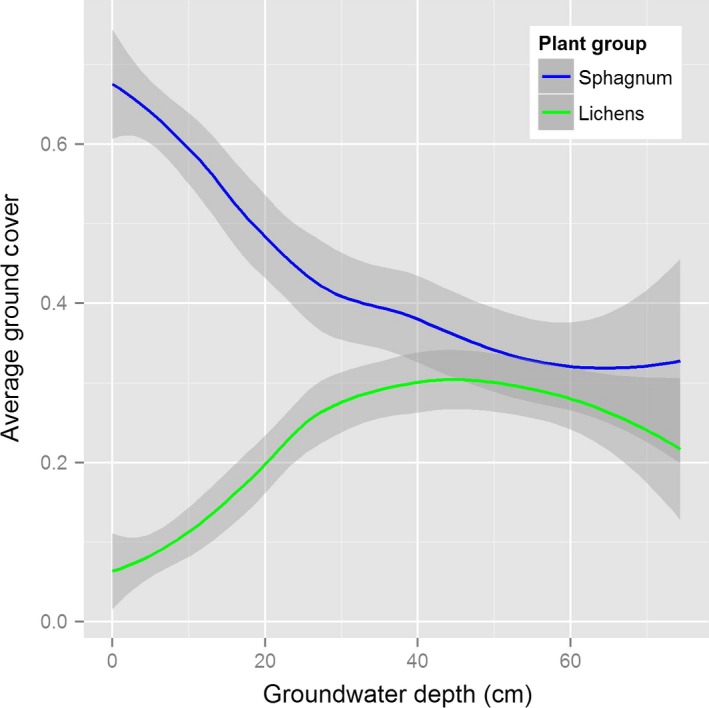
Relationship between *Sphagnum* mosses, terrestrial lichens, and groundwater depth in peatland ecosystems. The solid lines depict the mean smoother relationship using a generalized additive model, and the shaded areas depict 95% confidence intervals

The probability of observing zero terrestrial lichens increased in peatland conditions where groundwater is closer to the surface. Our data indicate that the ground cover of terrestrial lichens and *Sphagnum* moss is negatively correlated and shows opposing relationships with groundwater depth (Figure 2). Consistent with this result, terrestrial lichens are often found in drier, elevated bogs (Belland & Vitt, 1995; Nordbakken, 1996), while *Sphagnum* moss prefer to colonize over high, stable water tables with high soil water pressure (>100 mb; Price & Whitehead, 2001; Kangas et al., 2014; Appendix S3). The capillary fringe of organic soils is typically less than 15 cm (Price & Whitehead, 2001), and as a result, the soil surface is typically drier where groundwater depth is greater than 15 cm. Depth to groundwater can accordingly influence the types and abundances of nonvascular plants (e.g., lichens and mosses) that occur on the soil surface. Our results suggest that drier conditions (defined by deeper groundwater) promote lichens and wetter conditions promote *Sphagnum* moss. Small differences in groundwater depth (10–15 cm) can have a large effect on the types and abundances of nonvascular plants that occur in peatlands (Figure 2).

Terrestrial forage lichens were also significantly correlated with location covariates: northing and easting. Lichens were more abundant in the southern and western extents of the study area, and absence was more probable on transects positioned further east. These associations may be related to spatial conditions not encompassed by the other covariates or lichen dispersal and establishment mechanisms (e.g., wind dispersal of thallus fragments).

**Figure 3:**
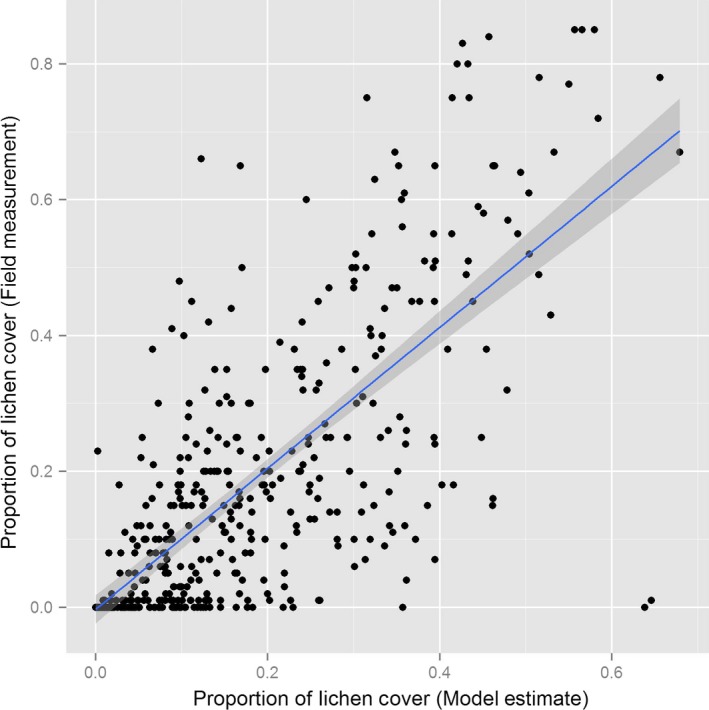
Relationship between the model estimate (fitted values) and field measures of the proportion cover of terrestrial lichens. The solid line depicts the mean linear relationship, and the shaded area depicts a 95% confidence interval

The residuals in the final model were approximately homogenous and normally distributed about zero with respect to sampling transect, latitude, and longitude, suggesting that errors in the final model were not correlated with these spatial effects. There was a significant (*p* < .001), positive linear relationship between our field measurements for terrestrial lichen cover and the fitted values from the final model (adjusted *R* ^2^ = .74). We recognize that the fitted values from the zero‐inflated beta cannot take a zero value (as zero measures were modeled as probabilities) and will accordingly overestimate a plant cover of zero. Still, the slope of the relationship was close to one (1.027; Figure 3) suggesting that the model has little bias and does not consistently over or under predict the cover of terrestrial forage lichens above a zero measure; the residuals in this relationship were otherwise homogeneous and normally distributed around zero.

**Figure 4:**
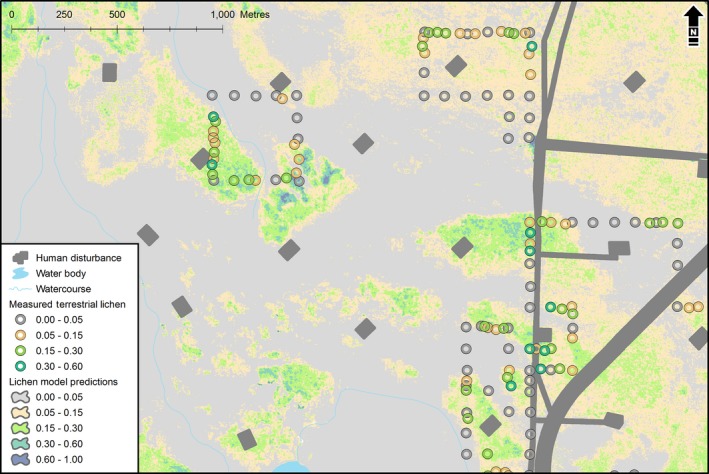
Plot of the estimated model for forage lichens predicted across a typical ecological extent within the study area (map interpolation)

The predicted model estimates that a large portion of the study area is defined by conditions that are poor for occupancy (i.e., high probability of zero) and contain low abundances of terrestrial forage lichens (Figure 4; 42% of the study area contains less than 1% lichen cover, and 61% contains less than 5% lichen cover). In addition, a large portion of the existing disturbance footprint does not overlap areas suitable for terrestrial forage lichen reclamation (Figure 4).

## 4 Discussion

Within Canada, the magnitude of woodland caribou population declines has been related to the amount of anthropogenic and wildfire disturbance within caribou ranges (Environment Canada 2012; Sorensen et al., 2008). The amount of disturbance within most ranges exceeds that associated with population growth and stability (Environment Canada 2012). Caribou conservation has accordingly placed an emphasis on caribou habitat recovery by reclaiming development footprints at rates faster than disturbance occurs. In the boreal forest, caribou habitats are often defined by unproductive peat ecosystems (Rettie & Messier, 2000; Schaefer & Pruitt, 1991) that require decades to recover naturally. Terrestrial forage lichens are no exception; they require three to eight decades to recover following disturbance (Kumpula, Colpaert, & Nieminen, 2000; Morneau & Payette, 1989). Innovative practices that can be used to conserve and restore woodland caribou habitats, including forage lichens, are needed.

Our results show that understanding both conditions related to a plant species abundance and occupancy (e.g., where a species does or does not occur) are important. We employed sampling and analytical approaches that enabled us to infer both of these conditions, improving our understanding of terrestrial forage lichen ecology and distribution relevant to reclamation management. For example, within boreal peatlands, terrestrial forage lichens are unlikely to occur (high probability of zero) under ecological settings defined by high vegetation productivity, a shallow water table, increased *Sphagnum* moss cover, and taller vegetation cover. Under these settings, reclamation practices are unlikely to be successful at recovering terrestrial forage lichens and could result in lost time and money. In contrast, under conditions of probable occupancy, reclamation is likely to be more successful if it emphasizes specific conditions defined by water table depth (>25 cm across the year); soil pH (3.5–5) and carbon–nitrogen ratios (>30); and vegetation composition (<35% *Sphagnum* moss cover, <10% deciduous shrub cover, and black spruce dominated canopy).

The conservation of woodland caribou habitat is identified as important across their distribution within the boreal forest. Our results provide an approach to help identify and delineate woodland caribou foraging habitat. The distribution and abundance of forage lichens have been poorly delineated within the boreal forest, largely because terrestrial forage lichens can occur within microsites that are difficult to identify at broad scales. As a result, woodland caribou habitat is often not specifically delineated in relation to forage lichens. Our study demonstrates that recent advancements in statistics and fine resolution remotely sensed data can be used to accurately identify the distribution and abundance of forage lichens.

The terrestrial lichen model estimated by this study is specific to the conditions within our sampling coverage and may not be applicable to other areas. The abundance of boreal vegetation species can vary over broad spatial extents due to climate, soils, and disturbance history (Thiffault, Grondin, Noël, & Poirier, 2015). For instance, previous inventories have shown that lichen abundance varies according to the distribution of wildfire history (Dunford et al., 2006; Morneau & Payette, 1989) and we constrained our sampling to ecosystems defined by a narrow range of wildfire history and forest age. Limiting this source of variation allowed us to infer ecological mechanisms, but may not be appropriate at coarse spatial scales where wildfires create a mosaic of forest ages. Rather, we have shown a sampling method, covariates, data resolution, and analytical method that can be used to accurately predict and estimate effects related to the absence and abundance of terrestrial lichens and other vegetation species. We employed data collection methods that are common to ecological studies and used an analysis method and statistical software package that is readily available to practitioners.

Vegetation abundance is often collected using proportional measures of cover, that is, Y∈(0,1). Ecologists have increasingly related vegetation cover to ecological characteristics using beta regression models (Korhonen, Korhonen, Stenber, Maltamo, & Rautiainen, 2007; Chen, Shiyomi, Hori, & Yamamura, 2008; Eskelson et al. 2011), which permit data bounded between zero and one with variances that increase in proportion to the mean. However, modeling proportional distributions that include zero and one values can be a challenge. Recent statistical development has extended the beta regression model (Ferrari & Cribari‐Neto, 2004) to include zero‐and‐one values (Ospina & Ferrari, 2010). Although some ecologists have used zero‐inflated beta distributions (Damgaard, 2009), to our knowledge, there have been few applications in ecology. Flexible platforms such as the R statistical environment are increasingly providing ecologists with methods to account for zero‐inflated data. Failing to account for zero‐inflation can lead to incorrect ecological inferences (Martin et al., 2005). Furthermore, estimating both abundance and the probability of zero can provide additional insight into ecological processes. We employed an inflated beta distribution model to improve our understanding of ecological phenomena and recommend its application to improve statistical estimation and inference in ecological studies on rare species, or where response variables are commonly defined by proportional values that contain zero and one.

## Conflict of Interest

None declared.
