# Supplementary material for: Estimating plant abundance using inflated beta distributions: Applied learnings from a lichen–caribou ecosystem
Source: Ecol Evol. 2016 Dec 20;7(2):486–93. doi: 10.1002/ece3.2625 (PMC5243790; doi:10.1002/ece3.2625)
Supplement: Supplementary file 1 [file ECE3-7-486-s001.docx]

**Appendix S1: Site conditions at lichen-abundant sites**

This study builds upon previous work we had conducted to understand lichen-abundant boreal ecosystems in a neighboring study area. We identified lichen-abundant sites using vegetation air-call protocols (Ministry of Forests and Range 2010). Survey crews visually estimated the proportion of terrestrial lichen cover, arboreal lichen class (Armleder, Stevenson & Walker 1992), and forest canopy characteristics at fixed sampling locations and opportunistic locations containing high lichen cover. A total of 90 sites were identified as having greater than 10% terrestrial lichen cover.

The following year, we returned to 83 lichen-abundant sites to better understand ecological processes that support high lichen cover in boreal ecosystems. Survey crews collected detailed vegetation data along a 30 m transect bisecting the site (Table S1). Subsamples of terrestrial lichens were harvested from each site to measure biomass. Soils data were collected at the start of each transect. Samples were taken from each soil horizon and submitted to a commercial laboratory (Exova Group Ltd.) where they were characterized for particle size, pH, total organic carbon (% dry weight), organic matter (% dry weight), and total nitrogen (% dry weight). We converted carbon and nitrogen content into a mass concentration per unit volume (mg/L) to facilitate comparing carbon-nitrogen ratios of mineral and peat soils.

Table S1: Description of vegetation and soils data collected at lichen-abundant sites

| Measurement | Description |
| --- | --- |
| Tree cover | Proportion of cover above 1.5 m by species, measured using a densitometer at 3 m intervals along the transect line |
| Tree age, height, and diameter | Age, height (m), and diameter at breast height (DBH) of a typical tree found in the dominant or co-dominant layer, measured using an incremental borer |
| Shrub cover | Proportion of cover measured using the presence of shrubs that intercepted each 1 m interval along the transect line |
| Ground cover | Proportion of cover of vascular species, nonvascular species, mulch, leaf litter, coarse woody debris, and water, measured within 50 cm x 50 cm vegetation plots placed at the start, center, and end of the transect line |

**Dominant terrestrial lichen species**

Terrestrial lichen cover was estimated at all 83 lichen-abundant sites, with 3 vegetation plots per site. Vegetation plots were located in both peat ecosystems (n=54) and mineral soil ecosystems (n=195). *Cladonia mitis* was the most common lichen species occurring in 96% of peat ecosystems and 93% of mineral soil ecosystems. *Cladonia mitis* was also the most abundant lichen species within vegetation plots, with a mean 40% cover in peat ecosystems and a mean 45% cover in mineral soil ecosystems.

Several other terrestrial lichen species consumed by caribou (Bergerud 1972; Schaefer & Pruitt 1991; Newmaster *et al*. 2013) were observed within vegetation plots, but less often and in lower abundances. *Cladonia* species other than *C. mitis* were observed in low abundance (mean cover <3%) in approximately one half of peat (53%) and mineral (46%) ecosystems. *Cladonia stygia* and *Cladonia stellaris* were observed in low abundance (mean cover <4%) but were less common in both peat (9 to 22%) and mineral (14 to 19%) ecosystems. Other terrestrial forage lichens that were observed in less than 10% of vegetation plots included *Cetraria nivalis*, *Cladonia rangerifera*, and *Cladonia crispata*.

**Arboreal lichens**

We estimated the correlation between terrestrial lichen cover and arboreal lichen class using our field data because both types tend to be found in older, unproductive forests. Beta regression revealed a positive relationship between arboreal lichen class and the proportion of terrestrial lichen cover (P<0.02, R^2^=0.43) indicating that, on average, terrestrial and arboreal lichens co-occur. The relationship between the two growth forms was stronger in uplands than in peat ecosystems, likely because high water tables in some peat ecosystems restrict terrestrial lichens while arboreal lichens can still persist.

**Forest canopy characteristics**

Lichen-abundant sites were characterized by mature, open-canopied black spruce (*Picea mariana*) and jack pine (*Pinus banksiana*). Sites were predominately located in stands between 50 and 95 years old. Median canopy closure was very low in peat ecosystems (15%) and moderately-low in mineral soil ecosystems (47%). Black spruce was common in peat ecosystems whereas jack pine was only observed at one peat ecosystem. In contrast, mineral soils contained either, or both, tree species. Sites had low tree heights (black spruce 2.5 to 14 m; jack pine 5 to 15 m) and diameters (black spruce 4 to 15 cm; jack pine 7 to 35 cm) given stand age, suggesting that forest productivity was particularly low in lichen ecosystems.

**Understory plant community characteristics**

The understory plant community was consistent in terms of species composition and low species richness in both peat and mineral soil ecosystems. Excluding lichens, the mean number of understory plant species was 10. Five understory plant species were observed at the majority of sites: velvet-leaved blueberry (*Vaccinium myrtilloides*), bog cranberry (*Vaccinium vitis-idaea*), big red-stem moss (*Pleurozium schreberi*), and Labrador tea (*Rhododendron groenlandicum*) were encountered in most mineral soil ecosystems; and bog cranberry, big red stem moss, Labrador tea, and *Sphagnum* mosses were encountered in most peat ecosystems. All five of these taxa are adapted to acidic soils with low nutrients (Longton & Greene 1969; Jeglum 1971; Ingestad 1973; Kurmis, Webb & Merriam1989; Bridgham, Updegraff & Pastor 2001); velvet-leaved blueberry and bog cranberry are often found in well-drained soils (Ritchie 1955; Vander Kloet & Hall 1981). Median shrub cover within lichen-abundant sites was low in both peat (4%) and mineral soil (10%) ecosystems.

**Soil Characteristics**

Lichen-abundant sites were primarily characterized by well-drained sandy soils or peat soils with poor soil fertility. Close to the soil surface (0 to 15 cm), where lichens establish, soils were acidic (pH of 3.5 to 5.0) and had high carbon-nitrogen ratios (30:1 to 80:1). Acidic soils contain high concentrations of H+ ions that are toxic to microbes and can limit root growth and nutrient absorption in vascular plants (Kidd & Proctor 2001). High carbon-nitrogen ratios tend to slow nutrient turnover, decomposition, and nitrogen mineralization by limiting soil microbes (Myrold 2005), and can suppress the productivity of vascular plants within peat ecosystems (Limpens, Heijmans & Berendse 2006). Maintaining low levels of nitrogen is likely important to lichens, as increased decomposition (Dorrepaal *et al*. 2005) and microbial activity (Bubier, Moore & Bledzki 2007) in peat ecosystems tends to coincide with increases in vascular plants. Overall, the high acidity and carbon-nitrogen rations indicate that lichens are primarily located in areas with low soil fertility. Mineral soil ecosystems had a median of 70% sand in the surface horizon; whereas peat soils were saturated with water (minimum depth where spaces in the soil were saturated with water) well below ground surface (>40 cm) indicating that both mineral and peat ecosystems tended to be well-drained near the soil surface for much of the year.

**References**

Armleder H.M., Stevenson S.K. & Walker, S.D. (1992). *Estimating the Abundance of Arboreal Forage Lichens*. Land Management Handbook, Field Guide Insert 7. British Columbia Ministry of Forests, Victoria, British Columbia.

Bergerud, A.T. (1972). Food habits of Newfoundland caribou. *Journal of Wildlife Management,* **36**, 913-923.

Bridgham S.D., Updegraff K. & Pastor, J. (2001). A comparison of nutrient availability indices along an ombrotrophic-minerotrophic gradient in Minnesota wetlands. *Soil Science Society of America Journal,* **65**, 259–269.

Bubier, J.L., Moore, T.R. & Bledzki, L.A. (2007). Effects of nutrient addition on vegetation and carbon cycling in an ombrotrophic bog. *Global Change Biology,* **13**, 1168–1186.

Dorrepaal, E., Cornelissen, J.H.C., Aerts, R., Wallen, B. & Van Logtestijn, R.S.P. (2005). Are growth forms consistent predictors of leaf litter quality and decomposability across peatlands along a latitudinal gradient? *Journal of Ecology,* **93**, 817–828.

Eskelson, B.N.I., Madsen, L., Hagar, J.C. & Temesgen, H. (2011). Estimating riparian understory vegetation cover with beta regression and copula models. *Forest Science,* **57**, 212-221.

Ingestad, T. (1973). Mineral nutrient requirements of *Vaccinium vitis-idaea* and *V. myrtillus*. *Physiologia Plantarum,* **29**, 239-246.

Kidd, P.S. & Proctor, J. (2001). Why plants grow poorly on very acid soils: are ecologists missing the obvious? *Journal of Experimental Botany,* **52**, 791-799.

Kurmis,V., Webb, S.L. & Merriam, L.C. (1986). Plant communities of Voyageurs National Park, Minnesota, U.S.A. *Canadian Journal of Botany,* **64**, 531-540.

Jeglum, J.K. (1971). Plant indicators of pH and water level in peatlands at Candle Lake, Saskatchewan. *Canadian Journal of Botany,* **49**, 1661-1676.

Limpens, J., Heijmans. M.M.P.D., & Berendse, F. (2006). Nitrogen in peatlands. *Boreal peatland ecosystems* (eds R.K. Wieder & D.H. Vitt), pp. 195-230. Ecological Studies, Vol. 188, Springer-Verlag, Berlin.

Longton, R.E. & Greene, S.W. (1969). The growth and reproductive cycle of *Pleurozium schreberi* (Brid.) Mitt. *Annals of Botany,* **33**, 83-105.

Ministry of Forests and Range. 2010. *Field Calibration Procedure for Photo Interpretation. Version 1.0*. Ministry of Forests and Range, Forest Analysis and Inventory Branch, Resource Inventory Standards Committee. Victoria, British Columbia.

Myrold, D.D. (2005). Transformations of nitrogen. *Principles and applications of soil microbiology* (eds D.M. Sylvia, J.J. Fuhrmann, P.G. Hartel & D.A. Zuberer), pp. 259-294. Prentice Hall, Upper Saddle River, New Jersey.

Newmaster, S.G., Thompson, I.D., Steeves, R.A.D., Rodgers, A.R., Fazekas, A.J., Maloles, J.R., McMullin, R.T. & Fryxell, J.M. (2003). Examination of two new technologies to assess the diet of woodland caribou: video recorders attached to collars and DNA barcoding. *Canadian Journal of Forest Research,* **43**, 897-900.

Ritchie, J.C. (1955). Biological flora of the British Isles: *Vaccinium vitus-idaea* L. *Journal of Ecology,* **43**, 701-708.

Schaefer, J.A. & Pruitt, W.O. (1991). Fire and woodland caribou in southeastern Manitoba. *Wildlife Monographs,* **116**, 1-39.

Vander Kloet, S.P. & Hall, I.V. (1981). The biological flora of Canada. 2. *Vaccinium myrtilloides* Michx. velvet-leaf blueberry. *Canadian Field Naturalist,* **95**, 329-345.
