# Supplementary material for: Estimating plant abundance using inflated beta distributions: Applied learnings from a lichen–caribou ecosystem
Source: Ecol Evol. 2016 Dec 20;7(2):486–93. doi: 10.1002/ece3.2625 (PMC5243790; doi:10.1002/ece3.2625)
Supplement: Supplementary file 2 [file ECE3-7-486-s002.docx]

**Appendix S2: Selection of terrestrial lichen covers by foraging caribou**

We explored the relationship between terrestrial lichen abundance and caribou winter feeding sites to determine if winter feeding areas were distinguishable from other locations based on terrestrial lichen abundance. Winter feeding sites of woodland caribou were identified in and surrounding the study area during the winters of 2008 and 2009 using cratering locations where caribou had removed snow cover to access forage (Photograph S2a). Cratering locations were observed during aerial ungulate surveys and ground-based snow tracking surveys conducted within the region; ecologists investigated a subsample of cratering areas to confirm caribou foraging activity. During summer, known foraging locations (n=30) and an independent sample of vegetation plots (n=220) were visited to measure terrestrial lichen and vegetation cover. Ecologists visually estimated the proportion of terrestrial lichen cover at known feeding sites and available vegetation plots. Visual lichen estimates were calibrated against lichen cover measurements collected along a 30 m transects line at a sub-sample of plots. During the summer survey, it was noted that not all black spruce dominated peatlands contained forage lichens and that the reason for their presence and absence was often visually obscure (Photograph S2b).

Photograph S2a. Examples of Caribou Crater Sites


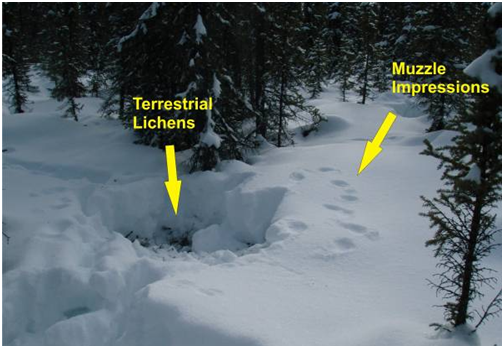

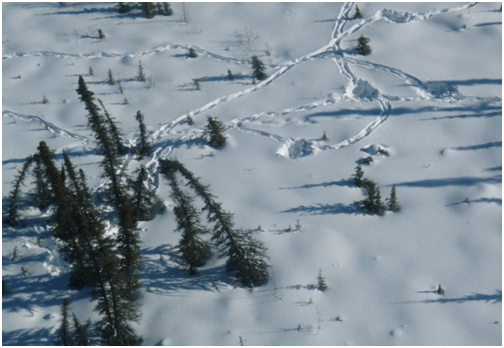


**Craters**

Photograph S2b. Black spruce peatland ecosystems with (a) and without (b) terrestrial forage lichens. Terrestrial forage lichens are depicted as white ground cover in photograph (a).


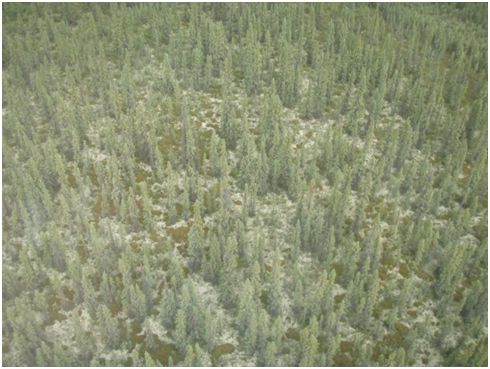

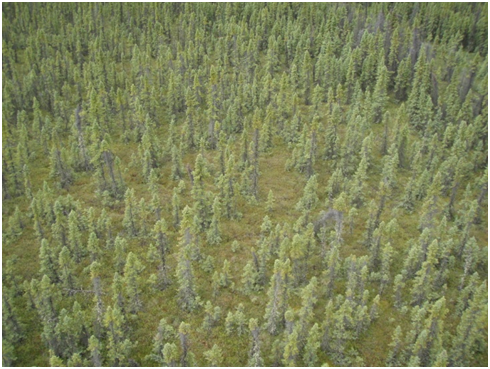


(a)_

(b)

We estimated a resource selection model for cratering locations using a use and available study design. Used locations were defined by terrestrial lichen cover at known foraging sites, whereas available locations were defined by lichen at vegetation plots. We considered two model forms: an exponential function that provides the relative probability of selection, and a logistic function that provides the actual probability of selection (Lele 2009). Both resource selection models were estimated using maximum likelihood methods (Lele & Keim 2006) with the final model form selected using Akaike Information Criterion (Akaike 1973).

The probability of winter caribou cratering activity was positively related to terrestrial lichen cover (P=0.008; Table S2). This result is consistent with woodland caribou in other parts of Canada where the probability of cratering activity increased markedly in relation to *Cladonia* cover and declined by only 0.05 as snow depth increased from 23 to 102 cm (Johnson, Parker & Heard 2000). Our analysis indicates that the probability of woodland caribou cratering activity is 0.50 at sites with 12% terrestrial lichen cover, and is 0.77 at sites with 25% terrestrial lichen cover (Figure S2). Overall, this analysis indicates that terrestrial lichen cover is an important component of caribou foraging activity in winter.

Figure S2. Logistic resource selection probability function model for terrestrial lichen cover at caribou forage sites. Filled circles represent the range of conditions where cratering activity was observed.


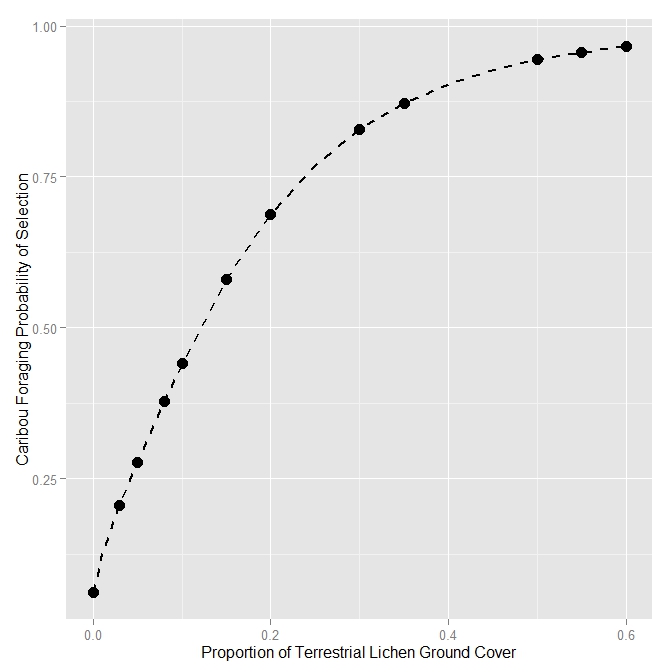


Table S2. Parameter estimates in the logistic resource selection probability function model for caribou cratering activity.

| Parameter | Estimate | Standard Error | z Value | Pr (>\|z\|) |
| --- | --- | --- | --- | --- |
| Intercept | -2.710 | 0.563 | -4.815 | <0.001 |
| Sqrt(lichen) | 0.783 | 0.295 | 2.656 | 0.008 |

**References**

Akaike H. (1973). Information theory as an extension of the maximum likelihood principle. *Second International Symposium on Information Theory*. (eds B.N. Petrov & F. Csaki). pp. 267‑281. Akademiai Kiado, Budapest.

Johnson, C.J., Parker, K.L. & Heard, D.C. (2000). Feeding site selection by woodland caribou in north-central British Columbia. *Rangifer,* **Special Issue** **12**, 159-172.

Lele, S.R. (2009). A new method for estimation of resource selection probability function. *Journal of Wildlife Management,* **73**, 122-127.

Lele, S.R., & Keim, J.L. 2006. Weighted distributions and estimation of resource selection probability functions. *Ecology,* **87**, 3021-3028.
