# Supplementary material for: Estimating plant abundance using inflated beta distributions: Applied learnings from a lichen–caribou ecosystem
Source: Ecol Evol. 2016 Dec 20;7(2):486–93. doi: 10.1002/ece3.2625 (PMC5243790; doi:10.1002/ece3.2625)
Supplement: Supplementary file 3 [file ECE3-7-486-s003.docx]

**Appendix S3: Spatial model of *Sphagnum*** **moss cover**

Our field data indicated that forage lichens and *Sphagnum* mosses were negatively correlated at 0.51. Including a covariate for *Sphagnum* moss cover (from collected field data) improved the fit of the terrestrial lichen model based on information criteria (AIC difference of -96.45). However, we desired the ability to extrapolate the forage lichen model on a map. We thus used our field measurements and spatial data to estimate a spatial covariate for *Sphagnum* moss cover that could be extrapolated on a map and considered in the inflated beta regression model estimating forage lichen cover. Although we did not record *Sphagnum* mosses to species at our sampling plots, our study area is typified by *Sphagnum angustifollium* and *Sphagnum fuscum*.

Field measures of *Sphagnum* moss cover included zero values and ranged between 0 and 0.95. To estimate a spatial covariate for *Sphagnum* moss cover we thus employed a zero-inflated beta regression model and used the same analytical methods as detailed for estimating the proportion cover of forage lichens (see methods in the main manuscript). The parameter estimates in the best fit model estimating *Sphagnum* moss cover are provided in Table S3. The predicted values from the estimated model and the field measures were correlated at R = 0.68.

Notably, the estimated model indicates that *Sphagnum* moss cover was negatively correlated with the groundwater depth covariate (Murphy *et al*. 2011), and that *Sphagnum* moss was more abundant when groundwater is nearer the surface (Figure S3). This relationship is consistent with the knowledge that *Sphagnum* mosses prefer to colonize over high, stable water tables, with high soil water pressure (>100 mb; Price & Whitehead 2001). In contrast, terrestrial lichens were most abundant at sites having groundwater depths greater than 15 to 25 cm below the surface (Figure 1D). Mechanistically, these contrasting groundwater associations likely contribute to the strong negative correlation between terrestrial lichens and *Sphagnum* mosses in boreal, organic soil ecosystems.

Table S3: Parameter estimates in the zero-inflated beta regression model for *Sphagnum* moss cover.

| Parameter | Estimate | Standard Error | t Value | Pr (>\|t\|) |
| --- | --- | --- | --- | --- |
| Beta Model (Proportion Model) | | | | |
| Intercept | 11.899 | 1.864 | 6.382 | <0.001 |
| Blue* | ‑0.110 | 0.017 | ‑6.593 | <0.001 |
| Near‑infrared* | 0.009 | 0.001 | 8.643 | <0.001 |
| Depth to groundwater | ‑1.003 | 0.191 | -5.240 | <0.001 |
| Slope | ‑0.186 | 0.055 | -3.377 | <0.001 |
| Sigma Link Function | | | | |
| Intercept | 1.484 | 0.067 | 21.992 | <0.001 |
| Slope | ‑0.146 | 0.076 | ‑1.907 | 0.057 |
| Logit Model (Zero‑inflation Model) | | | | |
| Intercept | ‑27.424 | 6.721 | ‑4.080 | <0.001 |
| Blue* | 0.213 | 0.058 | 3.670 | <0.001 |
| Near‑infrared* | -0.014 | 0.004 | -3.067 | <0.001 |
| Vegetation Height | 1.422 | 0.218 | 6.535 | <0.001 |
| Depth to groundwater | 2.685 | 0.760 | 3.533 | <0.001 |
| *Reflectance values taken from QuickBird imagery | | | | |

Figure S3. Plot of the fitted-values from the zero-inflated beta regression model estimating proportion cover *Sphagnum* moss against depth to groundwater. The plot depicts the marginal (net) effect of the depth to groundwater variable in the estimated model, which aids interpretation as there are multiple, partially correlated, variables in the model. The solid blue line depicts the mean relationship using a generalized additive model.


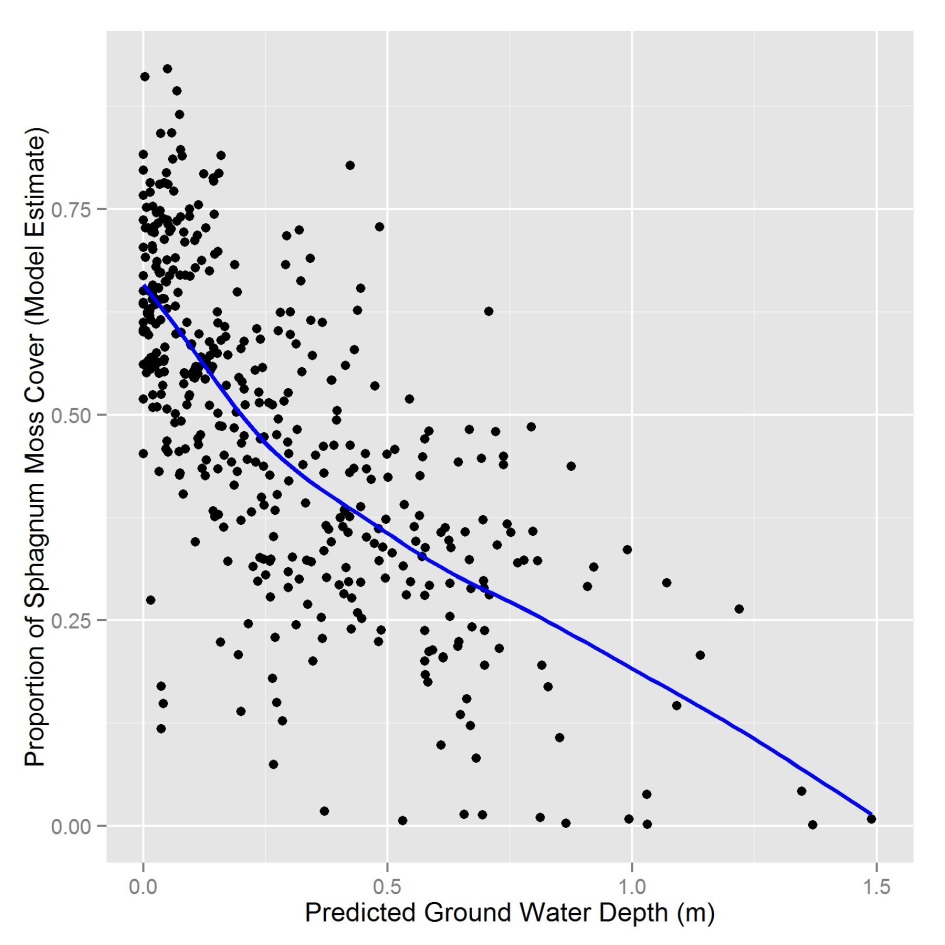


**References**

Murphy P.N.C., Ogilvie, J., Meng, F.R., White, B., Bhatti, J.S. & Arp, P.A. (2011). Modelling and mapping topographic variations in forest soils at high resolution: a case study. *Ecological Modelling,* **222**, 2314-2332.

Price, J.S. & Whitehead, G.S. 2001. Developing hydrologic thresholds for *Sphagnum* recolonization on an abandoned cutover bog. *Wetlands,* **21**, 32-40.
